# Exploring the solutions to the inherent perils of (the multitude of) guidelines – a focus group study of stakeholders’ perceptions

**DOI:** 10.1186/s12913-019-4261-4

**Published:** 2019-06-19

**Authors:** Louise H. K. Blume, Jamiu O. Busari, Nico J. H. W. van Weert, Diana M. J. Delnoij

**Affiliations:** 1 Medtronic GmbH, Meerbusch, Germany; 2 Department of Pediatrics, Zuyderland Medical Centrum, Heerlen, Netherlands; 3 Tilburg School of Social and Behavioural Sciences, Tranzo, Scientific Center for Transformation in Care and Welfare, Tilburg, University, Tilburg, Netherlands; 4 Department of Educational Development and Research, Faculty of Health, Medicine and Life Sciences, University of Maastricht, Maastricht, Netherlands; 5 Q! B.V, Nijmegen, Netherlands; 6 National Health Care Institute, Zorginstituut Nederland, Diemen, Netherlands; 7 Erasmus School of Health Policy & Management (ESPHM), Erasmus University Rotterdam, Rotterdam, Netherlands

**Keywords:** Guidelines, Hospitals, Implementation, External demands, Compliance, Standardisation, Requirements, Regulations, Stakeholders

## Abstract

**Background:**

Hospital boards have the responsibility to ensure compliance of hospital staff with guidelines and other norms, but they have struggled to do so. The current study aims to identify possible solutions that address the whole chain of guideline and norm production, use and enforcement and that could help hospital boards and management cope with norms and guidelines.

**Methods:**

We performed a qualitative study of three focus groups involving a total of 28 participants. In the third focus group, no new themes emerged, indicating that saturation was achieved. Focus group discussions were audiotaped and transcribed verbatim. Results were coded, and three themes emerged from the results. Thick description with selected key quotes is used to display the items in the result section.

**Results:**

In the first instance, norm developers, norm enforcers, and norm users acknowledged and reformulated the problem before they suggested solutions. The proposed concrete solutions, such as a clear description of the division of tasks within guidelines, clarity about the purpose of guideline recommendations, a maximum number of quality indicators for hospitals and implementation of an ensuring proper Information Technology (IT) infrastructure.

**Conclusions:**

This study aimed to find solutions for the problems that hospitals encounter in managing a multitude of norms and guidelines. Participants in this study acknowledged the fact that norms and guidelines have become difficult to manage at the hospital level and four potential solutions were identified.

**Trial registration:**

The study was retrospectively registered on the 21st of July in 2016 in the Dutch Trial Register as NL4061.

## Background

The use of guidelines and norms is supposed to provide clinical practice with the scientific basis that is required to justify the pursuit of consistent and safer health care delivery [1]. Even though evidence-based medicine (EBM) is the golden standard for decision making and has its distinct advantages [2, 3], an increasing number of disadvantages are also being observed [4]. Potential guideline users feel uncomfortable, over-controlled and angry due to regulators and inspections [5].

Apart from that, EBM has reached a point where the sheer volume of guidelines is a problem itself [6–8]. A conceivable strategy to address this problem will, therefore, be to reduce the disadvantages of implementing the guideline without losing the benefits. Achieving this goal, however, is particularly problematic in hospitals, where patients with innumerable (combinations of) diseases receive care, and which demands that employees and professionals from a variety of disciplines have to be familiar with guidance produced by many organizations [6].

Guidelines are part of the external demands that hospital boards, management, and staff are expected to comply with. In the Netherlands however, there are other norms to which hospitals have to adhere to [7, 9, 10], for example, obligations to publish quality indicator scores, consensus documents or laws and regulations. Applicable external demands include guidelines from non-clinical regulations and allied healthcare associations “such as standards, guidance, indicators, laws, rules, regulations, (volume and quality) norms from insurance companies, letters and reports from the inspectorate” [7, 10, 11]. In earlier studies, we focused on the strategies that hospital boards and managers adapt to cope with these demands. However, when it comes to compliance with guidelines and norms, there is only so much that hospital boards, managers, and staff can do (further referred to as hospitals). Some issues are beyond their sphere of influence. Therefore, in this study, we aim to look for solutions that address the whole chain of guideline and norm production, use, and enforcement. The system is larger than a hospital itself, and actors/stakeholders around it have a lot of influence on daily challenges a hospital faces. To increase the quality of healthcare, however, it is essential that stakeholders involved in guideline development, implementation and monitoring work together [4]. The barriers and possible interventions to the implementation of guidelines have already been summed up into three categories by Fischer et al. (2016). These include the “barriers related to physicians’ knowledge (e.g., lack of awareness and lack of familiarity), barriers that affect physicians’ attitudes (e.g., lack of agreement and lack of motivation) and those considered to be external barriers (e.g., patient-, guideline- and environment-related factors).” [12] Hence to be able to overcome these obstacles and to make possible interventions successful, norm producers, norm enforcers, and hospital boards need to make this a collaborate effort.

The question we, therefore, asked ourselves was: How can institutions that produce norms and guidelines for hospital care (e.g., patient organisations or professional associations of clinicians and nurses, guideline developers, trade unions and policymakers) and institutions that enforce these norms (e.g., the inspectorate for health care and insurance companies) contribute to improving the capacity of hospital boards, managers, and staff to improve care with guidelines?

This article focuses, on the one hand, on the interaction between these stakeholders and hospital boards. On the other hand, it focuses on norms and guideline processes: the whole chain of conception, dissemination, implementation, and enforcement. More specifically, we looked at:

- what developers of standards and guidelines for hospital care can do to reduce the number of guidelines/norms and improve clarity and consistency;
- what norm-enforcing institutions can do to focus and align priorities and reduce uncertainties for hospitals for which they are expected to comply;
- what hospital boards, managers, and staff can do to integrate norms and guidelines into hospital systems successfully.

This study aims to identify possible solutions that help hospitals to cope with norms and guidelines within hospitals, focusing on the external context. The findings might be useful for hospitals to handle norms and help policymakers to design better systems.

## Methods

We chose a qualitative research methodology for this study and carefully followed the Standards for Reporting Qualitative Research (SRQR) criteria to preserve the quality of this study [13].

We conducted three focus group interviews in the Netherlands to gather the information we needed to answer our research questions. Our rationale for choosing this method was to collect a lot of data from experts within a short period [14]. Furthermore, if the prior knowledge about a topic was sparse, the group dynamic could help extract precious data, as participants can actively think about existing processes and express new ideas.

### Participants

Focus group participants were recruited from parties in the healthcare sector using a purposeful sampling technique. The approach involved three steps namely inviting participants by email from trade associations national patient organizations, guideline development organizations, hospital board members, quality and safety managers and employees from hospitals, doctors, inspectorate and patient federation. Next, we distributed invitations through three online forums where possible participants were considered to be active (tertiary teaching hospital, the trade association for general hospitals, software vendors for Dutch hospitals) and finally, asked the invited participants to distribute the invitation to other participants of interest. Eligible participants had to be employed within the Dutch healthcare sector, speak Dutch and had to be impacted by guidelines in their day-to-day work. The participants represented four groups of stakeholder groups: “*norm developers*” (e.g. patient organisations or professional associations of clinicians and nurses, guideline development institutions, trade unions and policymakers), “*norm enforcers*” (e.g., the inspectorate for health care and insurance companies), “*norm users”* (hospital boards, professionals, managers, and staff) and “*policymakers*” (including researchers and politicians). The distribution of the stakeholders, the number of participants and the no-shows, are described in Table 1 (*N* = 28).

**Table 1:** Participants of the focus groups

	Number of participants and no-shows	Norm developers	Norm enforcers	Norm users	Researcher/consultant/ policymaker
Focus group 1	10 participants (12 invited, 2 could not attend)	3	2	1	4
Focus group 2	12 participants (12 invited)	4	1	7	0
Focus group 3	6 participants (10 invited, 2 could not attend, 2 no-shows)	2	1	2	1

In total, three focus groups were conducted in Utrecht in August and September 2016. After the first focus group, the moderator and researcher evaluated the process and decided that it was not necessary to adjust the topic list and general outline of the focus group. The second focus group generated the most relevant information. In the third focus group, no new themes emerged, indicating that saturation was achieved and refrained from conducting additional focus groups.

### Procedure

The participants received the invitation with three published articles [7, 10, 15] from the authors about hospitals and guideline implementation to create a common starting point for the discussion. They were asked to think about three questions (Table 2). At the beginning of each session, the moderator (DD) explained the purpose and procedures. This was done to ascertain that all participants had a similar level of knowledge about the topic, from where the discussion can start. The researcher (LB) was present during the focus groups but did not contribute unless clarification was required. The researcher audiotaped the focus group discussions and took notes during the focus groups. Participants were stimulated to express their opinions freely. They were informed that all identifying text would be anonymized before publication. Only the moderator, researcher, and participants were present during focus groups, and all participants signed an informed consent form. Each focus group lasted one and a half hours. The co-authors tested a semi-structured interview guide that was later used for moderating the focus group. The topics are shown in Table 2. It was determined beforehand that three or four focus groups could be conducted, but after three focus groups, data saturation was reached. The researcher transcribed the focus groups verbatim. Transcripts were not returned to the participants.

**Table 2:** Focus group semi-structured topics

Function/focus	Key questions
Questions on invitation	• What can developers of norms and guidelines for hospital care do to reduce the number of guidelines/norms and improve clarity and consistency? • What can norm-enforcing institutions do to focus and align priorities and reduce uncertainty for hospitals about what they are expected to comply with? • What can hospital boards, managers, and staff do to integrate norms into hospital systems successfully?
Hospital as part of the system	• What are possible solutions on a system level?
Norm developers	• What solutions can guideline developers put forward?
Norm enforcers	• What can norm enforcers contribute to a possible solution?
Stakeholders view	• Input from parties outside the hospital concerning experience problem by hospitals
Different perspective	• Possible solutions from a different perspective?

Three of the authors (JB, NvW, LB) independently read the transcripts thoroughly [1]. They coded the data openly and extracted key issues and underlying themes from the data. After that, the codes were ordered and checked [16]. Three themes emerged from the results, one theme having four sub-themes. All authors validated the themes. Thick description with selected key quotes is used to display the themes in the result section

## Results

In this section, the themes that emerged from the analysis of the focus group transcripts are presented. A total of three focus groups were conducted involving 28 participants.

### Acknowledging perceived difficulties

During the introduction of the focus group, the moderator explicitly explained that the problem of implementing guidelines in a hospital had been described in previous research (of which all participants had received the publications) and stressed that the aim of the focus groups was to look for solutions. Nevertheless, the participants from all categories once again acknowledged during all focus groups that the problem exists. They confirmed that there exists a sense of urgency to find solutions in hospitals and with stakeholders.

What you or they *[pointing to guideline developers]* release into the world is not a lot, but all of them together create a jungle (Norm user 8).

Participants emphasized the burden that perspectives are neither harmonized nor aligned. On the one hand, developers perceived that they support practitioners by developing guidelines and indicators, resulting in requirements owned by the sector. On the other hand, hospitals and professionals perceived those as a burden instead of support.

I, as a professional, am assisted if I can easily find and access things easily, as soon as I have a question (Norm user 4).

We develop the guidelines to assist professionals and patients, of course. If we disturb others with it, it is sad. We have to investigate together how we can find a good solution so that everyone is assisted (Norm developer 4).

Participants stated that hospitals want to provide excellent care, but norms can have an unintended (and maybe unwanted) impact. They felt that their resistance appeared from the plurality of musts/guidelines and as a result, the onerous process of having to implement the guidelines. All participants emphasized that the system of guidelines and norms did not assist professionals adequately and that it needed to be organized more effectively and efficiently. The Dutch Surgical Colorectal Audit makes information publicly available, leading to the following example of impaired effectiveness for quality improvement:

Anastomotic leakage in colorectal surgery is the indicator to assess whether you deliver good care or not. I am 100% sure that after we score poorly the first time, the surgeon does instruct the specialized nurse to look at it (*the indicator and therefore, the registration*) somewhat differently. Herewith, you completely miss your target. The indicator is used, in fact, to get a green checkmark. Based on this, healthcare contracts can be purchased! (Norm user 1).

Participants discussed that it is unclear which guidelines need to be followed and claimed that clear definitions are needed. Some referred to guidelines as quality improvement tools; however, others used them as enforcement tools.

We think that everyone must adhere properly to the rules and guidelines; the guidelines do not exist without a reason. But I can conceptualize that there is some need for more focus (Norm enforcer 2).

All participants acknowledged the perceived difficulties presented in the previous research, accentuating what their point of view is. Further citations are displayed in Table 3.

**Table 3:** Citations of participants reformulating the problem of guidelines

Citation	
‘People are not acquainted with it, they do not know it, they do not know what recommendations they have to know. It really depends on the interest of an individual professional whether it is used or not. I think that you need to look on system-level whether protocols are generally in line with guidelines. Quite often they are not even translated into practice. I think that it depends too much on the individual professional, and I think you should do much more on system level to implement it. And at the same time, I think, there is a problem with the system at organisational level.’	Norm developer 6
‘I did notice that there was a certain reluctance to reformulate an indicator, because the insurers may call them on account, and that was sensed immediately.’	Norm developer 9
‘Yes, if you look at the register, for example. We are asked to provide a tripartite now, including insurers. Thus, it is agreed that insurers play an even more important role. That was not the original question for guideline developers, by definition. Not because one is against it, but one looked at the content and how to deliver the best quality of care.’	Norm developer 2
‘I think it is a very difficult discussion, because I also realize what hospitals encounter. All parties awaken me to that. On the other hand, it is also true, that we have chosen a system in the Netherlands, where patients have an understanding of the quality in order to make the right choices. And yes, you will need information to do so.’	Norm developer 3
‘When I think of a quality label, I see it as a reward. I also notice that it happens in hospitals. Professionals say: we should keep this quality label.’ & ‘In psychology, not getting a reward is a punishment.’	Norm developer 3 & Norm user 6
‘It is questionable whether the field is really waiting for guidelines the way they are presented now.’	Norm user 8
‘And then you have the perverse incentives on all sides, and the control of the board of directors is fundamentally absent. Absolutely absent. There is no testing, nothing. For me, DICA is an example how it should not be done.’	Norm user 2
‘In the final phase, where you could start an improvement project, you cannot achieve it in practice, because you are hampered by so many factors. This is influenced by insurance companies, a patient, by available money or by management choices that must be made. So you have … What I am trying to say is that there is little room to establish improvement.’	Norm user 9
‘As a matter of fact, I would like to say that hospitals do want to provide good care. The resistance comes from the multitude and impossibility.’	Norm user 1
‘The challenge, therefore, is to provide the right information to the professional at the right time during the search. That is the big issue.’	Norm user 2
‘But a guideline, if I may call it that way, is a tool. It is an invitation. We, as a group, have determined that this is the best approach, and we can deviate from guidelines if we argue well. An indicator, on the other hand, encourages reflection, which stimulates the consideration: what is good for one specific patient, but not for the other?’	Norm user 1
‘… the whole exercise in the care sector was to deliver everything at one point in time for multiple purposes. But then you experience problems during realisation, as the insurers first said yes, but then they want to receive it at the first of October [*which is a different date than earlier agreed on*], because they need it for contracting. And then you have to work with the results from the previous year, so that is very difficult.’	Norm enforcer 1
‘The minute that all enforcing institutions, the patient, the insurers, and inspection, look over your shoulder in the doctor’s office, you might be more careful, perhaps you are going to make strategic choices instead of basing it purely on your professional expertise.’	Researcher/consultant/policymaker 5

### Concrete solutions

Besides several reformulations of the problem, the participants named exact solutions. The solutions formulated by the participants can be categorised into four categories.

#### Be clear about the target (group) and the imposed obligation

Most norm users and norm developers agreed that norm- developing institutions should stipulate the tasks for the organisation and professionals precisely in their guidelines. One attempt was described:

We try to indicate in the guideline whether the registration relates to the organization or the individual specialist, to achieve that the one in charge feels the responsibility for the registration. (Norm developer 2).

After the publication of a guideline, a hospital does not immediately undertake implementation action. They first decide which (part of the) guideline has priority. Participants suggested that certain criteria, such as risk reduction, quality benefits, and health benefits, should be mentioned in the guideline to contribute to a decision.

Can’t we do much more to highlight which things really make a big difference for the patient? (Norm developer 6).

Overall, participants underlined that distinction is needed between obligations and options as well as to whom they are relevant. One of the stakeholders from a norm- developing organization put forward a suggestion that his organization (and others) could follow.

Actually, you can say without difficulty: these are the guidelines having an organizational impact, and we will create an executive summary, and we sent the executive summary to every board of directors. That is only a small effort. We do not do it now, but it is one of the things we consider if that is what you are waiting for … You can also divide it into three categories: you can say this is purely professional, this is strictly organizational, and this is something in between. Then you are already on the right track. And if you do want to link this to a timeline, you can also highlight what is most important (Norm developer 5).

According to participants, this would make the process of implementation and sharing of tasks during execution much easier for users.

#### Be clear about the purpose of a norm/guideline/indicator

In each focus group, participants reported that norm developers should distinguish between different goals and targets of guidelines and indicators and the goals of publishing.

We can still improve a lot. The separation of the aim/purpose (Norm developer 6).

Several participants stated that norm developers could indicate that some indicators are used primarily for patient choice and are not explicitly intended to improve quality.

Not all indicators are intended to improve quality (Norm developer 3).

Participants stated that clear labelling of the purpose is desirable: which indicators are used to facilitate the choice for the future patients, which indicators are used for internal improvement, which indicators are used for contracts with insurers, etc. However, norm developers cannot control how their produced norms would be used or whether the norms are used for other purposes.

Guidelines are used by the inspectorate to enforce or by insurance companies for purchase, while the main objective is still reducing practice variation and knowledge transfer. However, the use by others is possible. Whether the use of the guideline turns out as intended or not, that is the question (Norm developer 1).

Measuring is important, as stated by several participants. One participant illustrated this with an example from 15 years ago, where the Netherlands and Belgium had different approaches for measuring MRSA:

Belgium had a long time no MRSA problem because they simply did not measure MRSA. Then you also ‘have no problem’ (Norm enforcer 1).

Participants stated that norm developers should indicate within a guideline the value and necessity for the guideline. Otherwise, users might not recognize the impact.

The usefulness and necessity of guidelines need to be explained so that people understand the underlying rationale of why they are doing something. ‘Yes, we need to do it for the board, or yes we need to do it for the inspectorate.’ That does not work. They need to understand what is useful and necessary (Norm enforcer 1).

#### Work with a maximum frame for indicators

In the first focus group, a discussion took place about norms, concerning public disclosure of quality indicator scores. Currently, Dutch hospitals are obliged to measure and publish about 1500 quality indicators. Participants agreed that quality indicators are useful but that a maximum number is required. Together, stakeholders should combine different indicators, according to the participants. After that, new ones can still be developed, but it can only be introduced after an old one gets erased.

Two years ago, we thought maybe we should use ONE indicator for multiple purposes. Then you limit the use of indicators, and then you can use the same outcome for several things. (Norm developer 3).

Participants specified that if different indicators are combined, the indicators should then only be used for the purpose for which they were created. Otherwise, the media and other parties could hijack the data. Participants reported that some attempts at synergy were already being made.

Insurers compiled a top 30, which is slightly different from the national top 30 which was worked with (Norm enforcer 4).

As explained by one participant, the first efforts are being made in the Netherlands: On a national level, 30 conditions were selected to improve the available information for patients with all parties, involving, among others, understandable guidelines for patients and the registration and publication of information. At the same time, insurers agreed that they would establish a limited number of quality indicators for 30 conditions.

#### Ensure proper IT infrastructure

The participants explored different solutions within the IT area in all three focus groups to make guidelines more usable for health care.

You should be facilitated. We now have the new electronic health record, and even though it was promised before we purchased it, the registrations [*referring to the registration of quality indicators*] are not included. Well, I think that we need to take big steps to facilitate the professional in this (Norm user 7).

They proposed that norm developers could provide guidelines in such a way that all of them could be found at the same spot. Meta-information and summaries, as well as implementation advisers, should be included. Hospitals should join forces to find an IT solution to connect guidelines to work sequences, to achieve that guidelines can be easily accessed at the point needed. Therefore, electronic patient devices should be linked to guidelines.

I think that the whole support by IT to our professionals is a challenge where we are in our infancy. And that these systems are simply not customised for our professionals yet. And I think that the people firing their systems at us, had too much to say so far, without us communicating clearly what we really need to make it work properly (Norm user 2).

The hospitals’ experienced dependence on the solutions electronic health record vendors provide. Participants proposed that hospitals should join forces negotiating with the electronic health record vendors, as implementation requires beneficial support at the particular time needed.

Well, the gap between IT in hospitals and the possibilities I have with this [*points at his mobile phone*], surprises me for years. (Norm user 4).

Participants suggested that norm-developing institutions could deliver guidelines and indicators in such a way that they are easy to integrate into the institutional IT support systems of users.

### Improvement of the system

The majority of solutions proposed by the participants targeted the problems in the existing system. The concrete solutions, for example, focused mainly on achieving changes within the current system and did not particularly suggest a different course. Additionally, they offer abstract ways to improve or name good examples from which one can learn. Participants referred to the system of the Dutch General practitioners as best practice, where all information needed is easily accessible via a website and a software application, without difficulties.

I think, that there is an international best practice of the Fins, again from the GPs, who did build the guidelines into their medical IT system. This initiative is from Finnish professional organizations. It is indeed an example of how they do it over there (Norm developer 6).

Participants stated that norm developers could increase transparency about how they publish and how they distribute guidelines.

We fool ourselves a little bit, as we are actually in a situation, I think, which is caused by us. By sharing little of what we do. I speak from a medical specialist’s perspective. Thus, you end up in a situation of increased mistrust (Norm developer 5).

Participants suggested involving managers and professionals: discover what they think is important and create more insight for them about the importance of implementation. After implementation, they proposed that managers and professionals should give feedback to norm developers about usability in practice.

And I think it is very sensitive to evaluate, as soon as it is fully developed and implemented, how it will be used in practice (Norm developer 8).

Participants recommended that the enthusiasm of doctors must be facilitated, to create bottom-up appetite/willingness and to address the relevance of the core of the guideline from the eye of the professional.

I would say that there needs to be willingness by the professionals to share information. You cannot enforce that externally. Make it a habit and necessity (Researcher/consultant/policymaker 5).

The solutions put forward by the participants aimed mainly at a change in culture, where it remained unclear who is responsible for what and when. They suggested finding alternative ways for hospital boards and managers to be in control. Additionally, they suggested strengthening the professionals.

You must create much more freedom in your system, to be able to work with local guidelines that are not enforced, but which are used to deliver the best care for the total patient population. That is the freedom that you need (Norm user 1).

What I do believe is in strengthening the professional, both the physician and the nurse (Norm user 1).

## Discussion

In this study, we looked for different possible solutions to help hospital boards, managers and staff to cope with norms and guidelines. While this focus group study specifically aimed for the identification of solutions, participants were not tired of repeating that norms and guidelines, including quality indicators, became unfathomable and unmanageable [7, 10]. Norm users, norm developers, norm enforcers and researchers/consultants/policymakers acknowledged the struggle of hospital boards and managers, described in previous research [7, 10, 15] by giving various examples and different interpretations of the problem. This observation is worthy of note, as the difficulties perceived by the norm users in previous research could have easily led to more defensive behavior by the other three groups of stakeholders. However, norm users experience guidelines as a burden instead of support because most guidelines are unavailable in forms that care providers can make use of when they need to make decisions at the bedside [17]. Hence, it was good to experience that the dialogue in the focus group led to various suggested solutions, which were supported by the stakeholders, no matter which role they played.

The results show that guidelines should state the target group of a recommendation and whether the implementation of this recommendation is obligatory or voluntarily. In doing so, judgment is required as recommendations have variable levels of evidence and vary in their impact on the quality of care when implemented. Apart from that, as norm enforcers are nowadays more likely to use guidelines during enforcement activities, there is a fine line between the ‘best practice’ and ‘standard of care,’ as described by an American study [18]. Our study shows that there is a need to set priorities, to be able to choose which (parts of) the many guidelines should be implemented first. Herewith, a helpful point of reference can be created for those responsible for priority setting in implementation, and to guide enforcement agents using guidelines as a basis for enforcement activities.

Different stakeholders value different aspects of quality of care, and therefore, many indicators are developed [19]. Indicators that are used to monitor the quality of hospital care are resource intensive, and one perceived barrier of using indicators is the lack of resources [20]. Increasingly, indicators are developed to increase transparency and enable patient choice. However, our results show that according to the participants it is not always clear for which purpose indicators are applied. To lower the administrative burden on hospitals, participants suggested agreeing on a maximum frame for indicators.

Our results show that the purpose of a norm/guideline/indicator needs to be clear. This is in line with the framework for guideline implementation, emphasizing that a stated goal of the guideline (e.g., clinical decision making, education, policy, quality improvement) may improve the actual use of the guideline [21, 22]. The motivation to comply is higher if the benefits of the guideline are highlighted [23]. Further research is necessary to understand if a clear purpose increases the actual use of the guideline.

Additionally, we need to fasten up the transformation to a new IT-directed guideline support system. The literature shows that the healthcare world is already busy with the enhanced use of computerized clinical guidelines [24]. Trivedi previously highlighted barriers and solutions back in 2002 [25], whereas our research shows that Computerised Clinical Guidelines are yet to be widely implemented in the Netherlands (besides in the General Practice). Research in other countries, such as Italy, shows that this leads to process-of-care improvements [26]. Since the transformation also costs money, there is a need for a business case.

The recommendation of ensuring proper IT infrastructure is not a novel result. The fact that it was mentioned so explicitly, however, stresses the importance of addressing this problem on a higher level than solely on the hospital level. Addressing IT usability during the development of guidelines could enhance the usability of norm users.

Throughout the focus groups, it became clear that hospitals needed to join forces and to become active partners in the process of guideline development. There was a consensus that such a collaboration would help to address and reduce obstacles to the use of guidelines at the national level. Regular consultations in an appropriate form between the parties could enhance the overall chain, and a feedback mechanism between the three parties could help to improve coherence. Further research on coherent improvements would be interesting.

Because of the magnitude and complexity of this problem, these issues could perhaps best be tackled in pilot projects on specific clinical topics, e.g. cancer care. Examples from solutions applied in other healthcare systems could offer guidance.

Interestingly, the suggested solutions were all directed at the current system and the chain of guideline production instead of a whole new course for guidelines use. The question was raised about whether guidelines are used for what they were intended, but there was no explicit discussion whether it is an appropriate tool for health policy in general [27].

### Study strengths and weaknesses

Focus groups have some inherent limitations, such as the participant selection, the contribution of outspoken individuals and the moderation of the discussion. We recruited stakeholders from various backgrounds and occupations. The moderator ensured contribution by all participants by starting with an introduction round to accustom all participants to talk in the group and finished with a round where participants could contribute their closing thoughts. A possible limitation is that the participants received three published articles with the invitation which might have influenced the discussion. However, because these articles had already been published, we suspected that a number of the participants in our focus groups had already read them. In order to create a common starting point for the discussion, we decided to share the publications with all participants.

Furthermore, some participants knew each other, since they are experts in the healthcare sector in the Netherlands. However, at the same time, it is a strength that the different parties discussed common objectives and possible improvements, as it had not happened elsewhere in this composition until these focus groups. Further research on possible solutions for guideline usage could provide clues for system changes to achieve improved quality of care.

The study was conducted in the Netherlands, which has a privately operated system based on regulated competition and with decentralized guideline development, but with centralized indicator development. Therefore, generalisability might be restricted. However, as other countries also struggle with increased guidelines and seek answers for: ‘Why are there so many guidelines? Are they all important, and how did we get here?’ [18].

## Conclusions

This study aimed to find solutions for the problems that hospitals encounter in managing a multitude of norms and guidelines. Participants in this study acknowledged the fact that norms and guidelines, including quality indicators, have become difficult to manage at the hospital level. Four potential solutions were identified, namely a clear description of the division of tasks within guidelines, clarity about the purpose of guideline recommendations, a maximum number of quality indicators for hospitals and implementation of an ensuring proper Information Technology (IT) infrastructure.

Representatives of hospitals should co-operate with other national agencies so that the four concrete solutions can be addressed collectively at the national level. In addition, they should actively put the need for alignment within the chain of guideline production, use, and enforcement on the policy agenda.

Participants believe that implementation is not only the responsibility of hospital boards and professionals and suggest that the distribution of the responsibilities for guideline implementation should be adapted to align with the needs and expectations of all the stakeholders in the process. If hospitals do not become an active player at the national level, we will continue muddling through, and that would be a waste of everyone’s effort.

## Data Availability

The datasets supporting the conclusions of this article are available in the DANS repository, 10.17026/dans-zd6-k6nj. Translated versions of the transcripts are available on reasonable request to the corresponding author.

## Abbreviations

EBM: Evidence-based medicine
IT: Information Technology
METC: Ethics committee
NVZ: Dutch Hospital Association
SRQR: Standards for Reporting Qualitative Research
